# Muscle-inspired bi-planar cable routing: a novel framework for designing cable driven lower limb rehabilitation exoskeletons (C-LREX)

**DOI:** 10.1038/s41598-024-55785-0

**Published:** 2024-03-02

**Authors:** Rajan Prasad, Marwan El-Rich, Mohammad I. Awad, Sunil K. Agrawal, Kinda Khalaf

**Affiliations:** 1 Department of Mechanical Engineering, Khalifa University, Abu Dhabi, UAE; 2 Health Engineering Innovation Center, Khalifa University, Abu Dhabi, UAE; 3 Department of Biomedical Engineering, Khalifa University, Abu Dhabi, UAE; 4 Khalifa University Center for Autonomous Robotic Systems (KUCARS), Khalifa University, Abu Dhabi, UAE; 5 Department of Mechanical Engineering and Rehabilitation and Regenerative Medicine, Columbia University, New York, NY USA

**Keywords:** Biomedical engineering, Mechanical engineering

## Abstract

There is a growing interest in the research and development of Cable Driven Rehabilitation Devices (CDRDs) due to multiple inherent features attractive to clinical applications, including low inertia, lightweight, high payload-to-weight ratio, large workspace, and modular design. However, previous CDRDs have mainly focused on modifying motor impairment in the sagittal plane, despite the fact that neurological disorders, such as stroke, often involve postural control and gait impairment in multiple planes. To address this gap, this work introduces a novel framework for designing a cable-driven lower limb rehabilitation exoskeleton which can assist with bi-planar impaired posture and gait. The framework used a lower limb model to analyze different cable routings inspired by human muscle architecture and attachment schemes to identify optimal routing and associated parameters. The selected cable routings were safeguarded for non-interference with the human body while generating bi-directional joint moments. The subsequent optimal cable routing model was then implemented in simulations of tracking reference healthy trajectory with bi-planar impaired gait (both in the sagittal and frontal planes). The results showed that controlling joints independently via cables yielded better performance compared to dependent control. Routing long cables through intermediate hinges reduced the peak tensions in the cables, however, at a cost of induced additional joint forces. Overall, this study provides a systematic and quantitative in silico approach, featured with accessible graphical user interface (GUI), for designing subject-specific, safe, and effective lower limb cable-driven exoskeletons for rehabilitation with options for multi-planar personalized impairment-specific features.

## Introduction

### Background

According to the World Health Organization (WHO), 15% of the world’s population, i.e., over a billion people, suffer from some kind of movement-related disability, mainly due to stroke, traumatic brain injury, as well as other neurological and musculoskeletal disorders^1^. Robotic rehabilitation has the potential to replace manual therapy^2,3^ due to its capability to offer automated intervention, quantitative measures for assessment and monitoring, higher intensity and repetitions during interventions, and the potential for home-based therapeutics and tele-rehabilitation. Robotic therapy also allows more accurate quantification of therapy duration, intensity/repetition, task, and patient-specific parameters, which can improve rehabilitation outcomes and optimize the process for both the therapist and the patient.

Three decades ago, the concept of modelling the lower limb as a pendulum was introduced through passive dynamic walkers^4^. Currently, most of the models used for lower limb exoskeletons are based on a rigid link model, either pendulum-like or a serial chain. Various devices such as C-ALEX^5,6^, ROPES^7^, C-LREX^8^, etc. employ an *n-link* pendulum-like model with user-specific inertial and length parameters to model the lower limb actuated by cables, where *n* is the number of links chosen. Most lower-limb robotic devices for stroke rehabilitation use direct actuation approach-based designs, where an actuator is mounted directly at the joint level on the user’s limb, along with rigid links^9–15^. However, these designs assume the knee joint as one degree of freedom (DOF) pin joint, which does not fully account for the biomechanics of the knee^16^ and may induce extra moments/reaction forces on the joint^17^, affecting user safety and comfort. These designs also add weight to the impaired limb, which already has compromised weight-bearing capacity and the ability to sustain additional inertial vibration. On the other hand, indirect actuation designs, such as those using cables exert moments on the joints based on an end effector approach, provide better compatibility with knee joint biomechanics and comfort to users.

Over the last decade, cable driven rehabilitation devices (CDRDs) have emerged as a viable solution for robotic rehabilitation, especially for the upper limb^18–23^. CDRDs have many advantages over other types of robotic rehabilitation devices, such as safety, low inertia and inertial vibration, lightweight design, high payload-to-weight ratio, large workspace, and modularity. Their modular configuration allows the targeting of movement dysfunction in multiple planes, as well as the facilitation of remote actuation consistent with the natural biomechanics of the joints. Additionally, CDRDs do not require exact alignment with physiological joints, reducing donning on/off time and enhancing safety.

While several CDRDs have been proposed for lower limb rehabilitation, such as C-ALEX^5,6^, ROPES^7^, and CORBYS^24^, most of them are limited to rehabilitation of motion in the sagittal plane^5–8,24,25,25–30^. This is because gait dysfunction in neurological patients, such as hemiparetic gait^31^, is mainly observed in the sagittal plane e.g. the reduction in hip/knee joint flexion/extension range of motion. However, hip circumduction in the frontal plane tends to be higher in stroke survivors, negatively impacting both their gait and balance. Current lower limb CDRDs typically do not incorporate out-of-sagittal plane motion (hip adduction) or allow for it passively in their physical design. Bi-planar cable driven devices have been mostly developed for upper limb rehabilitation^18–21^, with only a few addressing the lower limb^32^. In a previous work^33^, we proposed a novel 3DOF cable driven model for a lower Limb Rehabilitation Exoskeleton (C-LREX) which accommodates flexion/extension of the hip and knee joint, as well as, adduction/abduction of the hip joint. The model was able to successfully track bi-planar trajectory with 4 cables based bi-planar routing.

Cable-driven exoskeletons rely on cable tension to generate supportive joint moments, with cable routing being crucial for optimizing the system’s performance. However, this type of actuation requires adjustable cable tension and efficient routing to ensure the proper transmission of forces. The main disadvantage of cable-based actuation is the need for appropriate cable routing. The literature demonstrates various examples of cable-driven exoskeletons proposed, nevertheless, for a specific configuration or a specific routing^7,34,35^ only. Furthermore, the routings were not verified to ensure the capability to generate bi-directional joint moments, maintain safe clearance with the user body throughout the gait cycle, ensure no interference among the cables, and the intersection of the cables with hinges. The potential of leveraging routing configurations and optimal parameters has not been fully exploited in cable-driven exoskeletons, especially for clinical applications. To bridge the gap, this study presents a generalized framework and analysis of C-LREX with bi-planar routing based on a bi-planar model while ensuring the cable routing constraints. Additionally, the study extends the generalized framework-based analysis to identify the optimal routing parameters for a selected cable configuration. A MATLAB-based graphical user interface (GUI) application was created to make the proposed framework accessible to other researchers. Finally, the C-LREX model with optimal routing parameters is employed to track reference healthy trajectory with the impaired gait of a stroke survivor.

### Conceptual models and cable routing

To fully control the motion of an open chain cable-driven mechanism, the number of cables used must exceed the number of DOFs of the system being controlled^36,37^. Previous research^33^ has shown that a 3DOF model of the lower limb can achieve successful motion with as few as four cables. However, the routing configuration of the cables around the lower limb is crucial and presents one of the key challenges in cable-based transmission and actuation systems. To address this challenge, cable routing in CDRD can be inspired by the configuration of muscles in the human body, particularly those in the lower limb. Human muscles are grouped based on functional movement into unipennate muscles which move only one joint, and multipennate muscles which move several joints simultaneously. Some muscles have multiple attachment points, in addition to the origin and insertion, which increases the accuracy and flexibility of movement control. The complex muscle architecture in humans allows for the accurate and efficient performance of complex motions. The ability of multipennate muscles to span many joints and attach to multiple locations enables them to efficiently transmit forces and fluidly synchronize movement across many parts of the body. For example, the hamstring muscle in the lower limb is attached to the pelvis and the tibia bone and contributes to flexion of the knee joint as well as extension and rotation of the hip joint^38^, thus allowing for a variety of activities such as walking, jumping, and running.

**Figure 1:**
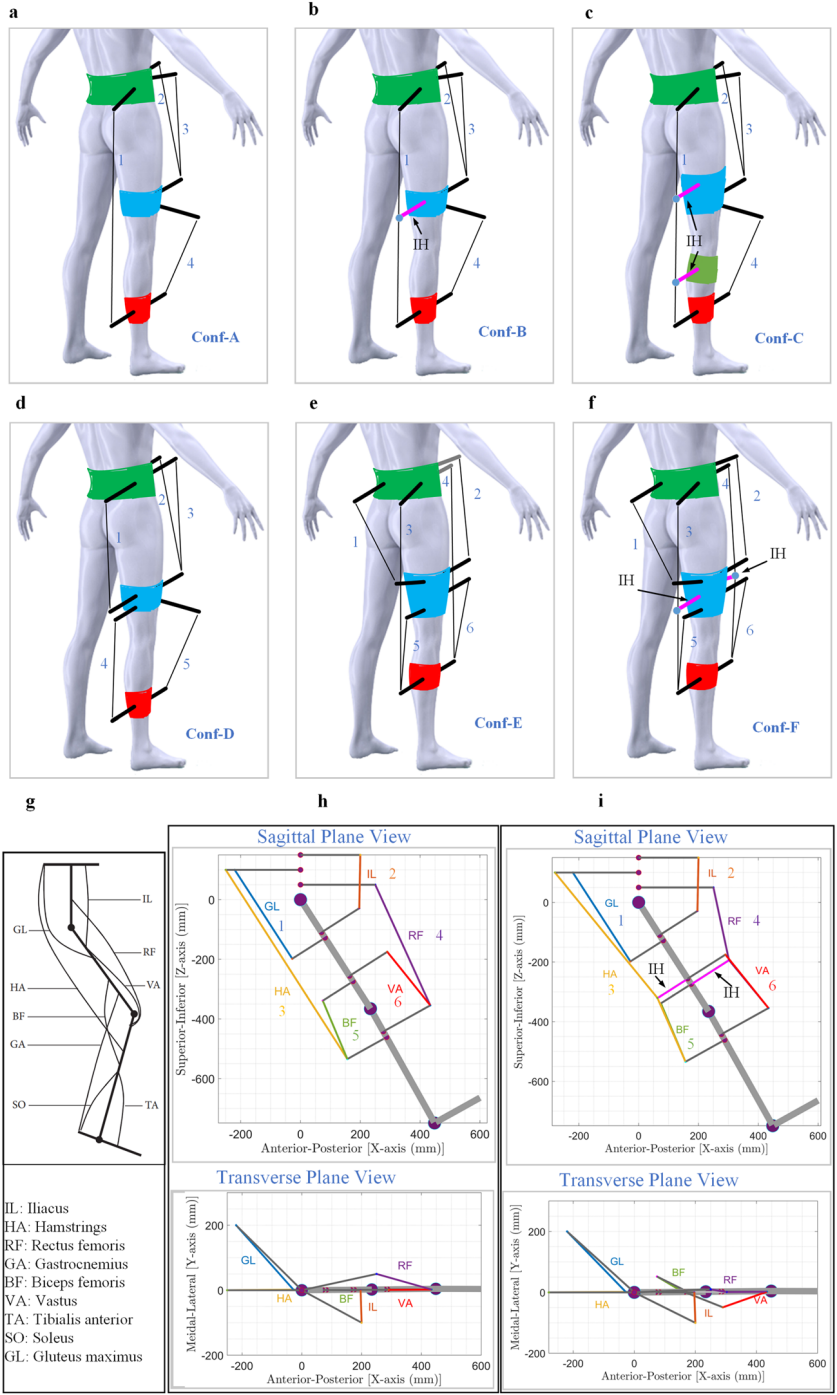
Conceptual models generated for analysis: (**a**). 4 Cable routing adopted from^33^ (Conf-A), (**b**). 4 Cable routing—I with one intermediate hinge for long cable 1 (Conf-B): IH refers to intermediate hinge, (**c**). 4 Cable routing—II with two intermediate hinges for long cable1 (Conf-C), (**d**). 5 Cable routing (Conf-D), (**e**). Biological muscle mimicked model (Conf-E), (**f**). Biological muscle mimicked model -I with one intermediate hinge for each long cable: 3 and 4 (Conf-F), (**g**). Major muscles contributing while walking^39^, (**h**). Sagittal and transverse plane views of biological muscle mimicked model (Conf-E), (**i**). Sagittal and transverse plane views of biological muscle mimicked model—I (Conf-F). Cables (IL and GL) are routed in the frontal plane, remaining cables are modified or slightly pushed in the frontal plane to avoid interference with the other cables.

Based on the concept of muscle routing, several routing configurations were generated using 4, 5, and 6 cables to track the desired reference trajectory in both the frontal and sagittal planes as shown in Fig. 1. In particular, the routing of long cables and the introduction of intermediate hinges were inspired by physiological muscle architecture. The cable configurations Conf-A, Conf-B, Conf-C, and Conf-D were routed from an engineering perspective. The objective was to achieve specific goals, such as creating a larger moment arm (by changing the cuff location), reducing the number of cables (by combining them), or using an antagonistic cable configuration (by employing two cables for each DOF). The 4-cable routing configuration is adopted from our previous work^33^. Routing configurations Conf-A, Conf-B, and Conf-C have a longer cable routed in the posterior zone which generates moments at both the hip and knee joints (dependent cable routing), unlike configuration Conf-D, where the cables generate moments at both joints independently. Furthermore, in configurations Conf-B and Conf-C, the long cable is guided to remain close to the limb via intermediate hinges, which allows sliding of the cables freely through itself, as shown in magenta color and indicated as IH in Fig. 1) at the thigh and shank levels.

Two configurations (Conf-E and Conf-F) cable routing mimicked the lower limb bionic-muscle routing. The major muscle groups contributing to walking^39^ are depicted in Fig. 1g. Since the lower limb was modelled with the foot fixed, the muscles associated with the motion of the foot are discarded. The actual cable routing of the biological muscle mimicked models (Conf-E and Conf-F) is depicted in Fig. 1h and Fig. 1i. Intermediate hinges were introduced to long cables to guide them close to the limb, similar to the multiple attachment points of lower limb muscles. In some conceptual configurations, the cuffs on the hip or thigh were rotated in the frontal plane to avoid interference and intersection with other cables/cuffs, or their lengths were adjusted. Each model was validated for cable routing constraints verification using the flowchart in Fig. 7c, and the routing was modified in case of failure to meet the constraints, such as bi-planar cuff orientation.

## Results and discussion

**Figure 2:**
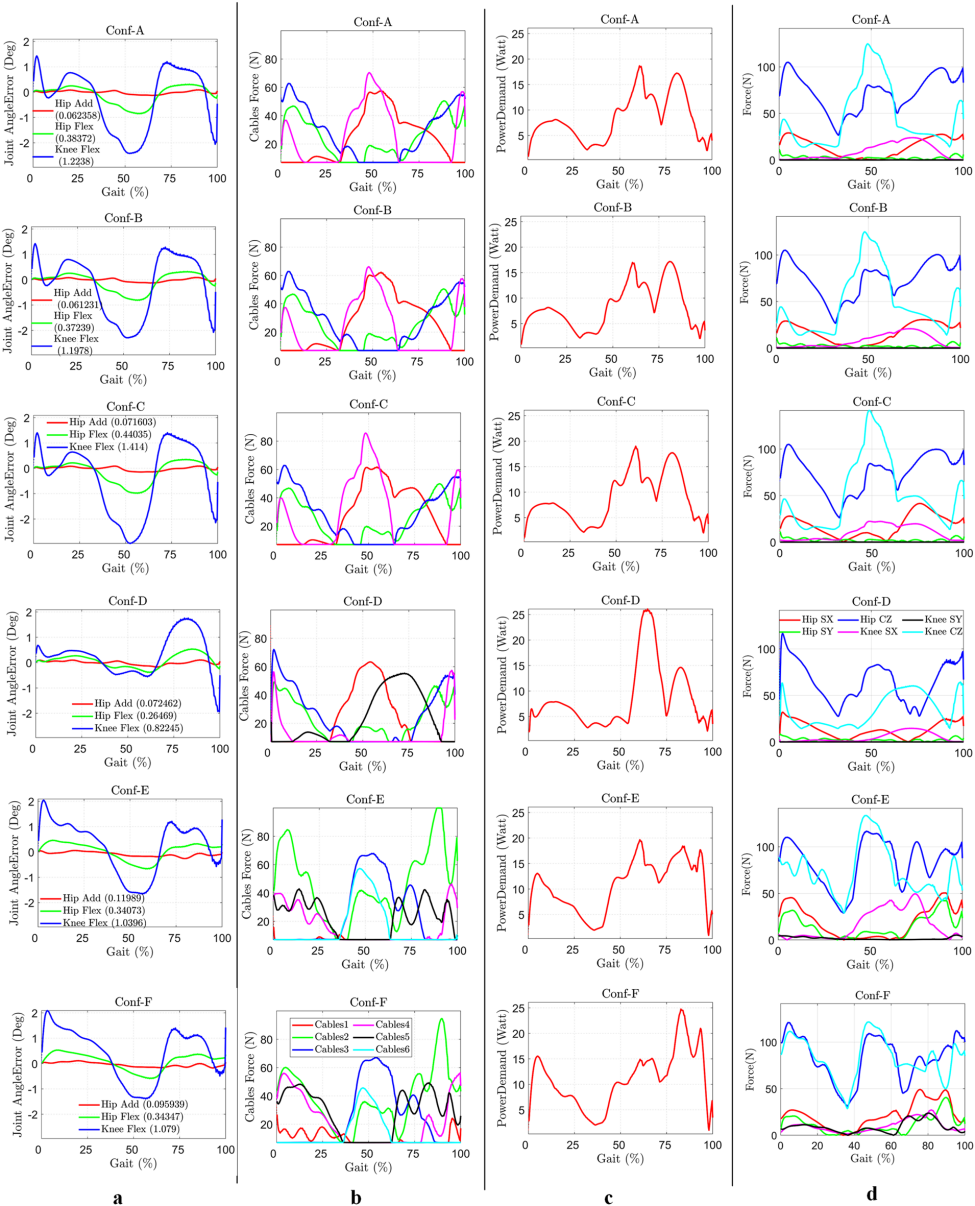
Simulation result analysis. (**a**), Error in the joint angle trajectory tracking (with RMSE in bracket). (**b**), Cable tension required to track the trajectory. (**c**), Power required (sum of power required through all actuator/motor). (**d**), Joint force components induced at the hip and knee joint due to applied cable tension [CZ: compressive force. SX and SY: anterior–posterior and medio-lateral shear forces respectively].

In the previous section, conceptual models were simulated using the 3DOF dynamic model over the gait cycle. The initial joint angle was assumed to be the same as the healthy reference based on the literature. All configurations, with and without intermediate hinges, successfully tracked the healthy trajectory as shown in Fig. 2a. Conf-D exhibited the least RMSE among all the configurations, with the least peak error in the knee flexion angle tracking. Conf-B exhibited the lowest peak cable tension requirement throughout the gait cycle, as shown in Fig. 2b. The biological muscle mimicked configurations (Conf-E and Conf-F) demanded the highest peak cable tensions.

The cable tensions applied to C-LREX generated moments on the joint, depending on the moment arm of the acting cable during the leg motion. The power requirement for each cable is represented in Fig. 2c, based on the applied cable tension and velocity. Furthermore, the applied cable tension induced joint forces (X, Y, and Z components shown in Fig. 6c) on the hip and knee joints, as depicted in Fig. 2d.

In Conf-A, Conf-B, and Conf-C, the cables spanning the knee joint were routed only in the sagittal plane, resulting in the medial–lateral component of the induced joint force (SY) being zero. Although the peak cable tension in Conf-D was not the lowest, it resulted in the least peak compressive force induced at the knee joint. This is likely due to Cable 4 in Conf-D being active only during the start and end of the gait cycle, which caused a significant drop in the compressive force induced at the knee joint. This may also be attributed to the routing configuration of the cable (number of cables and the origin-insertion points).

In this study, the engineering-inspired configurations generated higher knee joint compressive force compared to the compressive force generated at the hip joint, while the biological muscle mimicked configurations generated almost similar peak hip and knee joint compressive forces. These joint force components are the external forces that the user would experience while walking when assisted with C-LREX. A higher magnitude of such force components may result in discomfort to the user.

**Figure 3:**
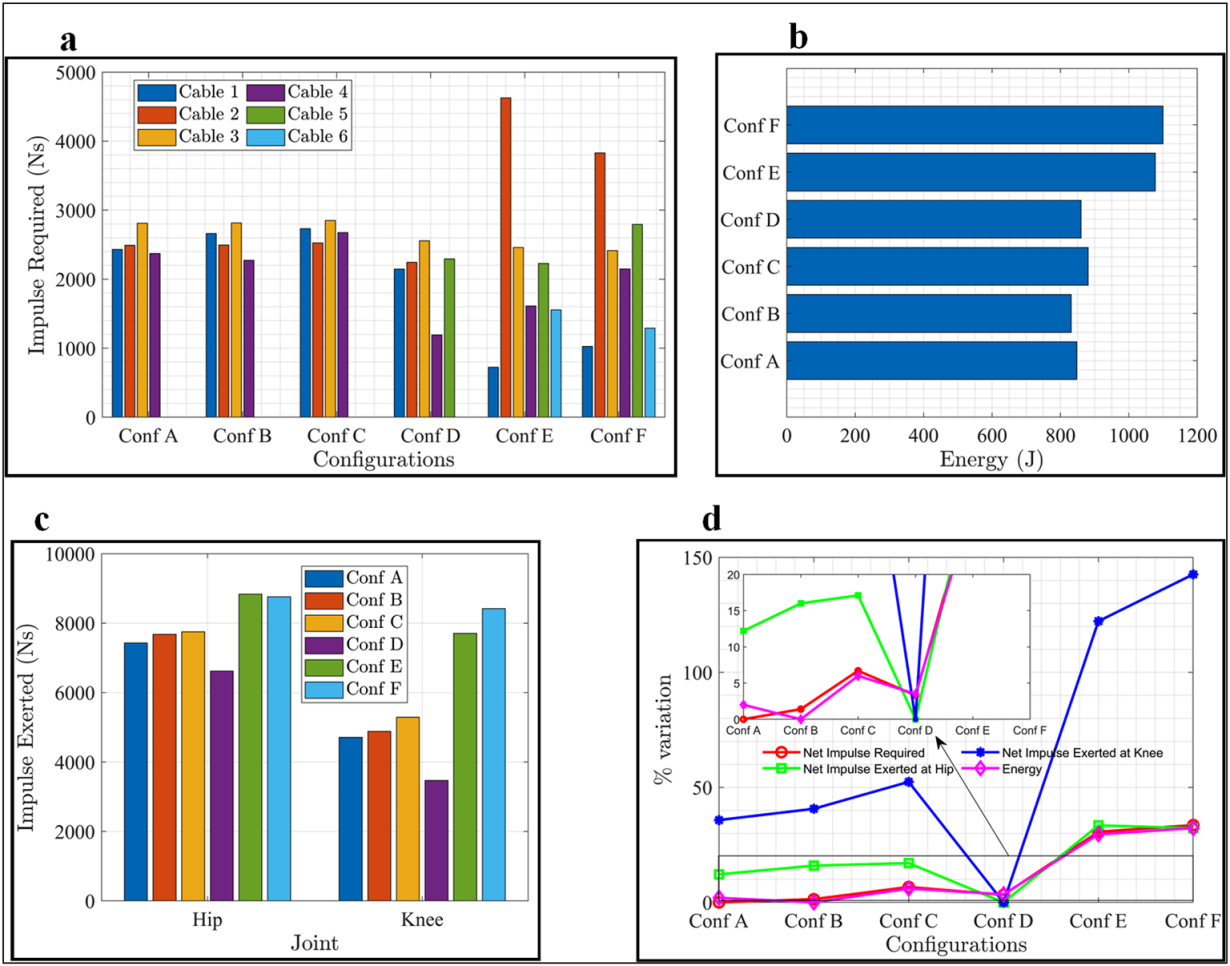
The area under output versus gait and variation analysis. (**a**) Area under cable tension versus gait time plot (Impulse required to C-LREX). (**b**) The area under power demand versus gait time plot (Energy requirement of C-LREX). (**c**) The area under resultant joint force induced at the hip and knee joint versus gait time plot (Impulse exerted to the user by C-LREX). **(d**) Percentage variation in various parameters (Estimated considering minimum as baseline): [Net refers to the sum of all cables and resultant for required and exerted scenario respectively].

The analysis focused on quantifying cable tension, power demand, and net-induced joint forces versus gait time plots. The impulse required by the C-LREX to alter the momentum of the lower limb, represented by the area under the cable tension versus the gait time, was evaluated (Fig. 3a). Conf-E and Conf-F exerted higher impulses on the lower limb joints due to higher joint force components over a longer duration during the gait cycle. Figure 3d depicted the variation in impulse and energy of each configuration relative to the lowest. Conf-A required the least impulse, while Conf-F required the highest impulse to generate the motion and momentum of the system.

The introduction of intermediate hinges generally increased the required impulse to the system, but peak cable tension increased in engineering-inspired configurations while decreasing in biological muscle mimicked configurations. Conf-B demanded the least energy with the least peak power requirement compared to others. Conf-E and Conf-F demanded the highest energy supply, while Conf-E and Conf-F exerted significantly higher impulses at the hip and knee joints than Conf-D. Conf-E exerted approximately 32% and 120% higher impulses at the hip and knee joints, respectively, while Conf-F exerted approximately 30 and 140% higher impulses at the hip and knee joints, respectively.

The biologically inspired routing configurations exhibited a relative underperformance compared to other configurations primarily because of the use of long cables. Long cables inherently offer less flexibility in generating individual joint moments independently, consequently demanding higher cable tension. This, in turn, results in larger tracking errors along and increased joint force component exertions.

### Identification of ‘suitable’ routing configuration

Based on the above decision metrics (requirements, performance, and exertion of forces), a weighted sum optimization function (as shown in the Eq. (10)) was formulated with different weights assigned to individual objective functions and their values along with the final values of the objective function are listed below in Table 1. The weights were selected to normalize the values of each objective function. This strategy was employed to standardize all objectives to a similar order of magnitude, irrespective of their units. Based on this, objective functions with higher orders of magnitude were prevented from exerting undue dominance over the optimization process. By assigning weights and normalizing values, our goal was to formulate an optimization problem, in which each objective function exerts comparable influence on the overall optimization process, ensuring that no disproportionate weight is assigned to one of these functions over the others. Currently, a fixed weight was assigned to each conceptual model corresponding to a specific objective function. This assignment was based on careful observation and evaluation of the objective function values, with the specific weight values *(λ*_*1*_*-λ*_*4*_*)* detailed in Table S-1. However, varying the weight of a specific objective function for individual conceptual models results in the prioritization of that specific objective function, consequently yielding distinct optimal solutions. Based on the results obtained through of the weighted sum optimization algorithm, we identified the “Conf-D” configuration, utilizing 5 cables, as a ‘suitable’ choice.

**Table 1:** Weighted sum optimization function values for configurations.

Configuration	Conf-A	Conf-B	Conf-C	Conf-D	Conf-E	Conf-F
Function Value *f1*	0.256	0.235	0.456	0.052	0.133	0.154
Function Value *f2*	0.031	0.032	0.035	0.028	0.046	0.043
Function Value *f3*	0.092	0.099	0.105	0.067	0.160	0.175
Function Value *f4*	0.087	0.084	0.094	0.090	0.141	0.147
Combined Function Value (*f*)	0.466	0.449	0.690	0.237	0.480	0.519

The cable routing configuration plays a crucial role in determining the performance and the requirement parameters of a system. In Conf-D, the cables were routed independently to generate hip and knee joint moments separately, resulting in minimum exertion of joint forces induced despite the slightly higher impulse requirement to the system due to a higher number of independently controlled cables. On the other hand, in biological muscle mimicked configurations Conf-E and Conf-F, two long cables [HA and RF (Fig. 1h,i)] generated dependent moments at both hip and knee joints. To counteract the uncontrolled generation of these moments, independent cable tension magnitudes were increased, and cable tensions had to be frequently varied to meet the requirements of both joints simultaneously. This resulted in a higher impulse requirement to the system.

Conf-C, which used additional intermediate hinges along long cable routings, had a higher peak cable tension requirement compared to Conf-A, leading to an increase in peak joint force components. However, Conf-E required a higher cable tension magnitude than Conf-F and exerted a lower magnitude of joint force components due to the introduction of intermediate hinges. The location and number of these hinges are crucial, as they guide the cables close to the limb, reduce the possibility of entanglement and vibration, and contribute to joint reactions at the support. However, depending on the location, the joint reactions could either be beneficial or detrimental to the system.

Investigations into bio-inspired cable routing for exoskeleton design have unveiled unforeseen challenges and limitations. While human muscles can generate high muscle forces (in the order of 1000’s N^40,41^), exoskeleton cables are restricted to a maximal applied tension of 100 N. Interference with the human body, including the joints and maximum cuff lengths, imposes additional constraints on cable placement, affecting the potential maximum joint moments that can be achieved^42^. Efforts to emulate the functionality of long muscles through extended cable routing have resulted in a limited ability to generate independent joint movements, contrasting with the efficiency observed in coordinated muscle synergies in human biomechanics. Moreover, the introduction of multiple attachment points in the cable routing system only partially alleviated this limitation. Conversely, an increase in the number of independently routed cables governing a singular joint demonstrated a positive correlation with improved model behavior. Specifically, Conf-D, characterized by cables autonomously controlling a single joint, outperformed configurations Conf-E and Conf-F, which emulated physiological muscles with two interdependent cable routings for each of the hip and knee joints. The integration of intermediate hinges along the cables exhibited marginal efficacy in enhancing overall system performance, contingent upon the quantity and spatial distribution of these hinges. In conclusion, our current findings underscore the inherent challenges in translating physiological muscle attributes to exoskeleton cable routing, emphasizing the need to explore alternative configurations, and highlighting the distinctive constraints associated with adapting bio-inspired solutions to exoskeleton design.

### Identification of actuator specification

The actuator’s specification can be estimated using the generalized framework based on the required cable tension and linear speed. The cables are driven by motors, which means that the specifications can be translated into the required motor torque and speed, assuming a suitable cable reel diameter. For instance, if a cable reel has a radius of 5 cm, the chosen Conf-D requires an actuator that can generate a torque of 4.5 Nm at a speed of 412 RPM. Figure S1 illustrates that, during the model simulation, the cable tension was limited to 100N, equivalent to a torque of 5 Nm.

### Estimation of optimal cuff location

The previous section identified a “suitable” configuration based on a weighted sum optimization function, which provided a general cuff location for cable routing. This section describes how to determine the optimal cuff locations for the cable routing in Conf-D, which has a total of 45 variables for cuff locations (9 cuffs, each defined by 5 parameters). Determining the optimal cuff locations systematically is time-consuming due to a large number of variables, so a Monte Carlo simulation approach was adopted. A predetermined number of random datasets (configurations) from a range of values for variables was selected (Figure S2a), and the model was simulated (Fig. 8a). A weighted sum optimization problem was then formulated, and the dataset with the minimum function value was selected as the optimal configuration (Fig. 8b).

**Figure 4:**
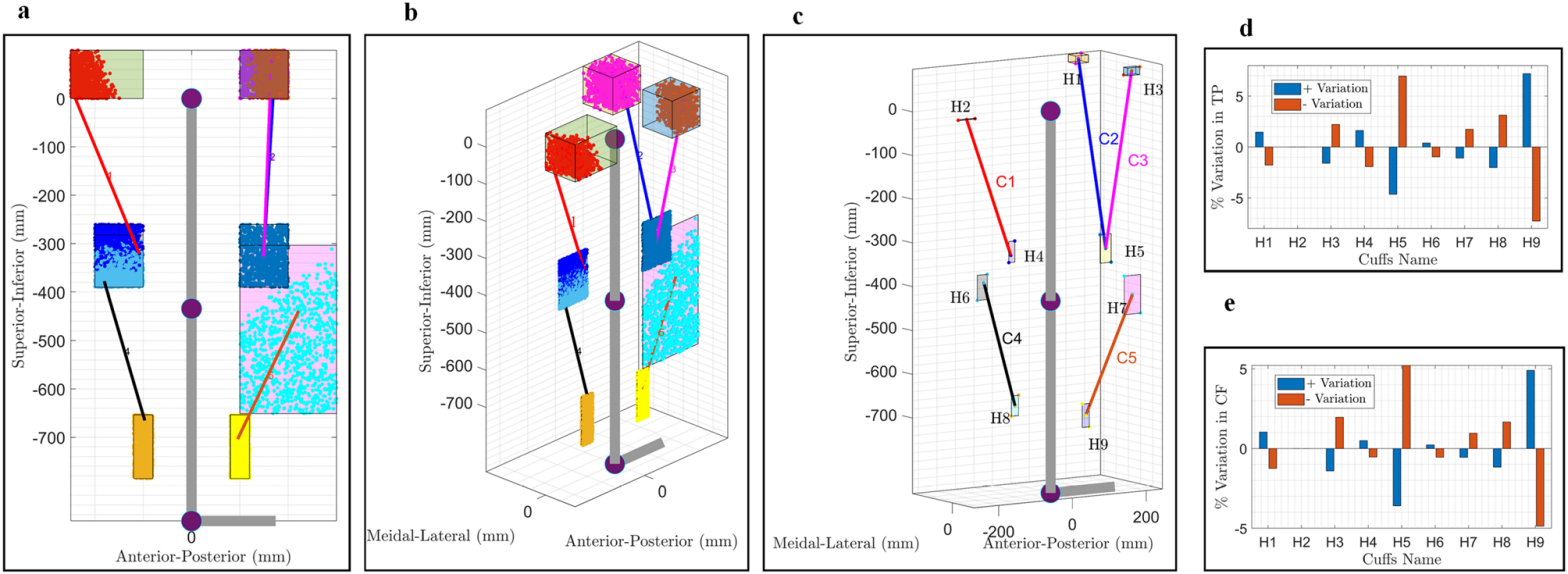
C-LREX with 5 cables configuration (Optimal Conf-D). (**a**). Sagittal plane view. (**b**). Isometric view (Specified cuff zones shown in colored rectangular boxes, feasible cuff prediction shown in dots, optimal cable routing shown in colored lines). (**c**) Optimal cuff location with allowable variation ranges (C represents Cable, H represents Cuff hinges). (**d**) Influence of variation in the cuff length on tracking performance (TP). (**e**) Influence of variation in the cuff length on weighted sum optimization cost function. In the figure legend, ± variation refers to variation in cuff location.

To reduce the number of variables during the optimization process, the angles $t\_lh$ and $ft\_lh$ (Fig. 6c) were set to a fixed value, and corresponding values $a\_lh$ and $fa\_lh$ were assigned so that the cuff end location is measured along axes (Figure S2b). The range of values for each variable is shown in the colored rectangular zone in Fig. 4a,b. During the Monte-Carlo simulations, 22,086 random samples were analyzed to yield 1000 verified samples for the healthy reference trajectory. The obtained optimal cable cuff locations (Fig. 4a,b) are specific to the selected gait speed, reference joint and angle trajectory, user anthropometric data, and constraints imposed during routing constraints verification. Nevertheless, the framework is open to variation in these parameters. During simulation, 27 samples failed to meet the routing constraints verification, resulting in 973 simulated models. The optimal routing parameters were obtained via a weighted sum optimization function as stated in the Eq. (10) and the inclusion/exclusion of new functions in the multi-objective optimization routines may alter the optimal configuration. Further analysis with the obtained optimal configuration and base configuration of Conf-D can be performed, similar to the approach adopted for the estimation of suitable configuration, to quantify the improvement in performance along with the minimization of requirements.

In physical exoskeleton development, it is difficult to place the cuffs exactly at the same points obtained via the optimization process. Thus, the influence of cuff position variation on the overall system performance (weighted sum optimization cost function value) and tracking performance was analyzed. The optimal cuff locations were varied by ± 10% in the position of the cuff with respect to the origin in both the positive and negative directions along axes, creating a small cubical zone ($x \pm \Delta x,y \pm \Delta y,z \pm \Delta z$) of variation, or rectangular zone for the sagittal plane bounded cuffs. The optimal cuff location of the origin of cable 4 (H6) and the insertion of cable 1 (H4) was varied by only ± 5% since a ± 10% change violated the cable routing constraint verification, as shown in Fig. 4c). It was found that such variation does not significantly influence the overall performance, while cuff hinges H5 and H9 were more sensitive to variation compared to H2 (Fig. 4d).

### Tracking healthy reference trajectory with stroke gait employing C-LREX

**Figure 5:**
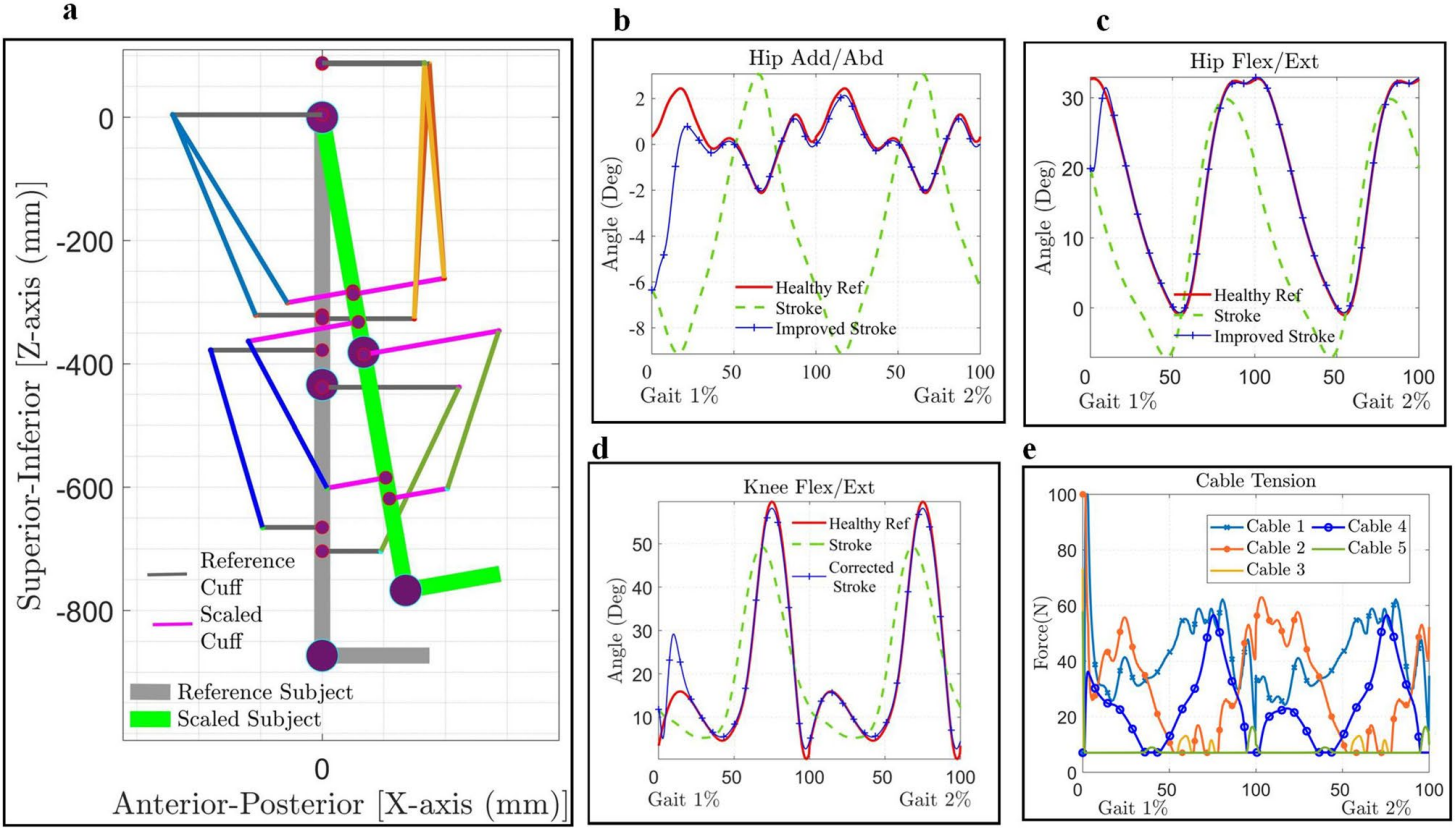
Tracking healthy reference trajectory with stroke gait via C-LREX Conf-D. (**a**) Scaling of optimal cable routing to another subject (the cuffs were scaled only for the distance along the superior-inferior axis). (**b**–**d**). Joint angle tracking with bi-planar stroke gait. (**e**) Cable tension applied to track healthy reference trajectory for selected the stroke gait.

The optimal cuff locations for the 5-cable driven model of C-LREX (Conf-D) were estimated considering the passive elastic joint moments of the limb only during the gait cycle. However, in reality, some active joint moments would also be exerted by the user depending on the nature and degree of gait impairment. To evaluate the effectiveness of C-LREX in assisting bi-planar impaired gait, the 5-cable driven model of C-LREX (Conf-D) was used with the estimated optimal cuff location. The user’s joint active moment was estimated using Eq. (1) based on the kinematics of the impaired gait and the anthropometric data of the user and used in addition to the passive elastic joint moment by the model. It was also assumed that the stroke limb could cover the healthy limb’s range of motion (workspace). The identified optimal cuff location was parameterized based on the user’s anthropometry (limb segments’ length) for scaling (*c_lh* along the Z axis as shown in Fig. 5a.) to allow for adaptation to different users. The gait trajectory of a female stroke survivor measuring 160 cm in height and 55.8 kg in weight was recorded at Cleveland Clinic Abu Dhabi (CCAD). The model was simulated for two gait cycles to analyze the influence of the transition from one gait cycle to another.

The model successfully forced the impaired gait to track the healthy reference trajectory (Fig. 5b–d). The initial deviations were higher as the model starts tracking from the stroke gait and assisted it to reach the healthy reference gait. During the transition from one gait cycle to another, the model tracked more closely, and the deviations vanished since the limb was already in motion. The average cable tension remained below 65 N (Fig. 5e). A higher cable tension requirement was observed at the beginning of the gait cycle due to the higher deviation and absence of initial limb velocity. The peak initial cable tension requirement was not observed during the transition of gait. The peak-induced joint force components remained below 105 N (maximum observed for hip joint compressive force SZ).

### Limitations

The current cable-driven exoskeleton model, like others in literature, has limitations because it only uses cable tension as a constraint. This limitation arises because the actuator design and specifications depend on both torque and speed. To address this issue, future work could include an objective function based on cable velocity and add cable velocity as an additional constraint. Additionally, the optimal cuff location obtained in this study is dependent on user-specific anthropometric data, gait trajectory, walking speed, and controller parameters. Although this approach allows for a personalized simulation, there is a need to develop automated algorithms to accommodate subject-specific variations. The current framework also lacks information on passive joint moments in the frontal plane during hip adduction, necessitating the inclusion of more quantitative physiological data in future studies. If contractures or spasticity are impending the ability to achieve a gait cycle close to healthy reference trajectory, our model is open to integrate user-provided data regarding the restrictive moments resulting from spasticity or contractures during gait, which can be predicted by a quantitative model. This user-provided information empowers our model to assist in aligning the impaired trajectory as closely as possible with the healthy reference trajectory, while respecting the constraints of the system. In practical situations, the model will consistently apply joint moments that compensate for the joint angle discrepancies caused by spasticity or contractures.

## Conclusions

Cable driven lower limb exoskeletons have multiple advantages over link-driven exoskeletons, including lightweight, remote actuation, negligible inertia, and inertial vibration, as well as modular configurability. The main disadvantage of cable-based actuation systems is the need for appropriate cable routing. To address this, we proposed an in-silico systematic framework for quantitatively analyzing cable routing configurations and architectures for CDRDs. The framework accommodates bi-planar cable routings with a constraint-driven routing verification approach.

Firstly, several configurations based on bi-planar cable routing, inspired by muscles and muscle routing architecture, were analyzed to identify a ‘suitable’ cable configuration. The results indicated that cable routing is a key parameter that dictates the performance and requirements of the cable configuration. Dependent cable routing demanded a higher impulse to change the momentum of the system and also exerted a higher impulse to the joint due to more applied cable tension. Routing long cables via intermediate hinges, similar to biological muscle multi-point attachments, could improve the overall performance, depending on the number of hinges and locations. Secondly, the optimal routing parameters (cuff locations) were estimated for a selected configuration for a specific gait cycle, anthropometric data, and control parameters. Lastly, the optimal routing-based C-LREX configuration is simulated to assist a stroke survivor's gait to track a reference healthy trajectory. In the current framework, the model only imposes cable tension as a constraint. To constrain the cable configuration of a CDRD, such as C-LREX, within specified actuator (motor) specifications, velocity has to be included as a constraint to the model, which will be explored in future work.

## Materials and method

### 3DOF lower limb model

**Figure 6:**
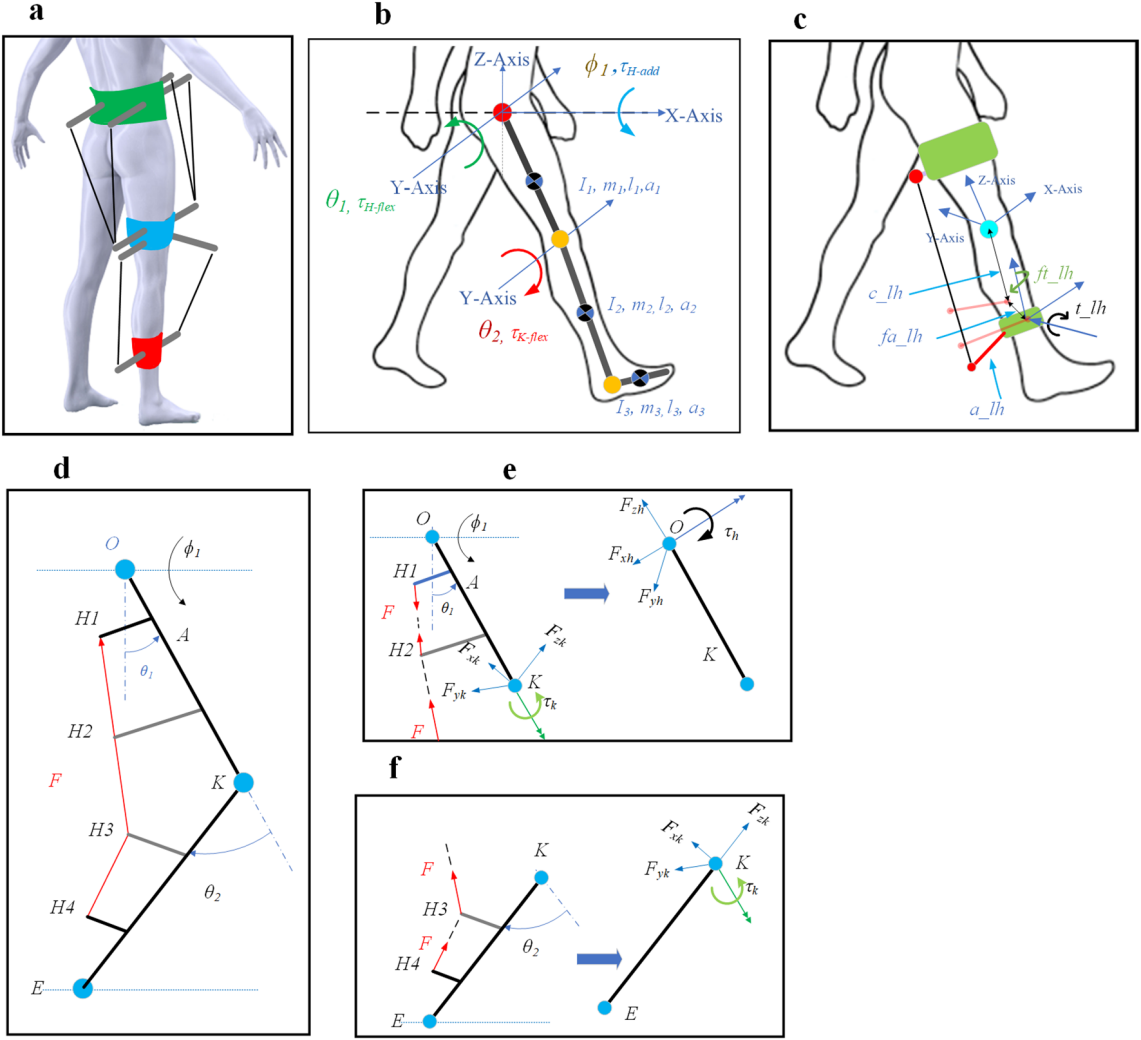
Modeling of C-LREX. (**a**) Conceptual model of C-LREX. (**b**) 3DOF Model of the lower limb for C-LREX^33^. (**c**) A generalized cuff definition is shown for Shank Zone as a reference. (*c_lh*, and*fa_lh*refer to the distance of the cuff end from the physiological joint center along the Z-axis, Y-axis refer to the distance of the cuff end from the physiological joint center along the Z-axis, Y-axis, *a_lh* refers to the length of the cuff, *t_lh* and *ft_lh* refer to rotation of the cuff about Y-axis and Z-axis). Cable force to joint moment mapping: (**d**) Two link models with cable tension applied from thigh to shank with intermediate hinges between insertion and origin of the cable, (**e**) Equivalent joint moment and component forces acting at the hip joint, (**f**) Equivalent joint moment and component forces acting at the knee joint. H2 and H3 are intermediate hinges that only guide the cable.

In our previous work^33^, we developed a model for the C-LREX exoskeleton based on 3 degrees of freedom (DOF) *n-*link pendulum model of the lower limb. The model included the motion of the hip and knee joints in the sagittal plane (flexion/extension) and the hip in the frontal plane (adduction/abduction) as shown in Fig. 6 a and Fig. 6 b. We ignored the rotation in the transverse plane as it is less significant during walking.

We used Euler’s—Lagrange formulation to derive the dynamic model with the generalized coordinate $$q = [\begin{array}{*{20}c} {\phi_{1} } & {\theta_{1} } & {\theta_{2} } \\ \end{array} ]^{T}$$, where the hip abduction/adduction angle and the hip and knee flexion/extension angles are represented as $\phi_{1}$, $\theta_{1} \,and\,\theta_{2}$, respectively. The dynamic equation can be written as:

1 $$M\left( q \right)\ddot{q} + C\left( {q,\dot{q}} \right)\dot{q} + G\left( q \right) = \tau$$

where *M(q)* represents the inertial matrix (3 × 3), $C\left( {q,\dot{q}} \right)$ represents the Coriolis component (3 × 1), *G(q)* represents the Gravitational components (3 × 1) and $\tau$ represents the torques on the joints (3 × 1) and is represented as : $$\tau = \left[ {\begin{array}{*{20}c} {\tau_{H - add} } & {\tau_{H - flex} } & {\tau_{K - flex} } \\ \end{array} } \right]^{T} \in {\mathbb{R}}^{3 \times 1}$$.

We assumed that the foot is fixed to the shank at a perpendicular orientation, and we developed the model only for the swing phase of the motion for simplicity. The C-LREX exoskeleton is powered by cables that are directed through specialized cuffs. To include these cuffs in the mathematical analysis model, we parametrically defined them using a 5-parameter definition, as shown in Fig. 6c. Each cuff is locally defined with the nearest biological joint as a reference point.

It is essential to note that the joint moment in Eq. (1) results from the combination of the applied cable tension and the passive elastic joint moment contributed by the limb. When using the C-LREX during walking, only passive elastic joint moments in the sagittal plane, as detailed in reference^43^, we taken in consideration. The contribution in the frontal plane was excluded due to the absence of available data on joint passive moments in that plane in the literature.

To generate the required joint moment, the C-LREX exoskeleton uses collective cable tensions that contribute to either the hip joint or knee joint, or both joints simultaneously, depending on the lower limb kinematics at the instant of motion, cable routings, and the tension applied. The joint moment can be estimated using the vector projection principle as similar to work^8^. We estimated the Jacobian for each cable using the vector projection method^8,44^, and then combined them to obtain the control performance matrix, which represents the collective conversion of cable tension to equivalent joint moments.

We introduced additional hinges between the insertion and origin of the cable to vary the moments being generated. The unit vectors along the limb sections were estimated (along the X, Y, and Z axes) using the joint angles of the lower limb and are dependent on the geometric configurations. The cable tension vector is projected on the limb unit vectors to estimate the equivalent joint force components (Fig. 6d–f). The equivalent joint moment is obtained by the cross-product of the distance vector and cable tension vector.

The reaction at each hinge (*H*_*1*_*, H*_*2*_*, H*_*3*_*, H*_*4*_) is estimated as:

2 $$\begin{gathered} \overset{\lower0.5em\hbox{$\smash{\scriptscriptstyle\rightharpoonup}$}}{{R_{4} }} = \,\overrightarrow {{F_{{H_{4} H_{3} }} }} = F \cdot \hat{u}_{{H_{4} H_{3} }} \hfill \\ \overset{\lower0.5em\hbox{$\smash{\scriptscriptstyle\rightharpoonup}$}}{{R_{3} }} = \,\overrightarrow {{F_{{H_{3} H_{2} }} }} - \overrightarrow {{F_{{H_{4} H_{3} }} }} = F \cdot \left( {\hat{u}_{{H_{3} H_{2} }} - \hat{u}_{{H_{4} H_{3} }} } \right) \hfill \\ \overset{\lower0.5em\hbox{$\smash{\scriptscriptstyle\rightharpoonup}$}}{{R_{2} }} = \,\overrightarrow {{F_{{H_{2} H_{1} }} }} - \overrightarrow {{F_{{H_{3} H_{2} }} }} = F \cdot \left( {\hat{u}_{{H_{2} H_{1} }} - \hat{u}_{{H_{3} H_{2} }} } \right) \hfill \\ \overset{\lower0.5em\hbox{$\smash{\scriptscriptstyle\rightharpoonup}$}}{{R_{1} }} = \,\overrightarrow {{F_{{H_{1} H_{2} }} }} = F \cdot \hat{u}_{{H_{1} H_{2} }} \hfill \\ \end{gathered}$$

For the shank, assuming $\hat{u}_{SH - x,} \hat{u}_{SH - y,} \hat{u}_{SH - z}$ are the unit vectors, the joint force components and joint torque acting at the knee are estimated as:

3 $$\begin{gathered} \overrightarrow {{F_{zk} }} = F \cdot \left( {\left( {proj_{{\hat{u}_{SH - z} }} \hat{u}_{{R_{4} }} } \right) + \left( {proj_{{\hat{u}_{SH - z} }} \hat{u}_{{R_{3} }} } \right)} \right) + = F \cdot \left( {\left( {\left( {\hat{u}_{SH - z} \cdot \hat{u}_{{R_{4} }} } \right)\hat{u}_{SH - z} } \right) + \left( {\left( {\hat{u}_{SH - z} \cdot \hat{u}_{{R_{3} }} } \right)\hat{u}_{SH - z} } \right)} \right) \hfill \\ \overrightarrow {{F_{yk} }} = F \cdot \left( {proj_{{\hat{u}_{SH - y} }} \hat{u}_{{R_{4} }} } \right) + \left( {proj_{{\hat{u}_{SH - y} }} \hat{u}_{{R_{3} }} } \right) \hfill \\ \overrightarrow {{F_{xk} }} = F \cdot \left( {\left( {proj_{{\hat{u}_{SH - x} }} \hat{u}_{{R_{4} }} } \right) + \left( {proj_{{\hat{u}_{SH - x} }} \hat{u}_{{R_{3} }} } \right)} \right) \hfill \\ \,\,\overrightarrow {{\tau_{k} }} = \left( {\overrightarrow {{KH_{4} }} \times \left( {F \cdot \hat{u}_{{R_{4} }} } \right)} \right) + \left( {\overrightarrow {{KH_{3} }} \times \left( {F \cdot \hat{u}_{{R_{3} }} } \right)} \right) \hfill \\ \end{gathered}$$

where, $\hat{u}$ is the unit vector and the subscript denotes the direction of the unit vector. *F* is the magnitude of the applied cable tension, $\overrightarrow {{F_{xk} }} ,\overrightarrow {{F_{yk} }} ,\overrightarrow {{F_{zk} }}$ are the joint force components acting in the x, y, and z axis respectively at the knee joint, $\overrightarrow {{KH_{3} }} ,\overrightarrow {{\,KH_{4} }}$ are the position of the hinges with respect to the knee joint, and $\,\overrightarrow {{\tau_{k} }}$ is the joint moment acting the knee joint.

Similarly, the joint force components and joint torque acting at the hip joint can be estimated. The joint component forces estimated for the shank also contributed to the joint component forces acting at the hip joint. Assuming $\hat{u}_{TH - x,} \hat{u}_{TH - y,} \hat{u}_{TH - z}$ are the unit vectors for the thigh section, the joint reaction and the moment vectors can be estimated as:

4 $$\begin{gathered} \overrightarrow {{F_{zh} }} = \left( {proj_{{\hat{u}_{TH - z} }} \overrightarrow {{F_{zk} }} } \right) + \left( {proj_{{\hat{u}_{TH - z} }} \overrightarrow {{F_{yk} }} } \right) + \left( {proj_{{\hat{u}_{TH - z} }} \overrightarrow {{F_{xk} }} } \right) + F \cdot \left( {\left( {proj_{{\hat{u}_{TH - z} }} \hat{u}_{{R_{2} }} } \right) + \left( {proj_{{\hat{u}_{TH - z} }} \hat{u}_{{R_{1} }} } \right)} \right) \hfill \\ \overrightarrow {{F_{xh} }} = \left( {proj_{{\hat{u}_{TH - x} }} \overrightarrow {{F_{zk} }} } \right) + \left( {proj_{{\hat{u}_{TH - x} }} \overrightarrow {{F_{yk} }} } \right) + \left( {proj_{{\hat{u}_{TH - x} }} \overrightarrow {{F_{xk} }} } \right) + F \cdot \left( {\left( {proj_{{\hat{u}_{TH - x} }} \hat{u}_{{R_{2} }} } \right) + \left( {proj_{{\hat{u}_{TH - x} }} \hat{u}_{{R_{1} }} } \right)} \right) \hfill \\ \overrightarrow {{F_{yh} }} = \left( {proj_{{\hat{u}_{TH - y} }} \overrightarrow {{F_{zk} }} } \right) + \left( {proj_{{\hat{u}_{TH - y} }} \overrightarrow {{F_{yk} }} } \right) + \left( {proj_{{\hat{u}_{TH - y} }} \overrightarrow {{F_{xk} }} } \right) + F \cdot \left( {\left( {proj_{{\hat{u}_{TH - y} }} \hat{u}_{{R_{2} }} } \right) + \left( {proj_{{\hat{u}_{TH - y} }} \hat{u}_{{R_{1} }} } \right)} \right) \hfill \\ \overrightarrow {{\tau_{h} }} = \left( {\overrightarrow {OK} \times \overrightarrow {{F_{zk} }} } \right) + \left( {\overrightarrow {OK} \times \overrightarrow {{F_{yk} }} } \right) + \left( {\overrightarrow {OK} \times \overrightarrow {{F_{xk} }} } \right) + \left( {\overrightarrow {{OH_{1} }} \times \left( {F \cdot \hat{u}_{{R_{1} }} } \right)} \right) + \left( {\overrightarrow {{OH_{2} }} \times \left( {F \cdot \hat{u}_{{R_{2} }} } \right)} \right) + \overrightarrow {{\tau_{k} }} \hfill \\ \end{gathered}$$

where, $\overrightarrow {{F_{xh} }} ,\overrightarrow {{F_{yh} }} ,\overrightarrow {{F_{zh} }}$ are the joint force components acting in the x, y, and z axis respectively at the hip joint, $\overrightarrow {OK} ,\overrightarrow {{\,OH_{1} }} ,\overrightarrow {{\,OH_{2} }}$ are the position of the knee and hinges (H1 and H2) with respect to the hip joint, and $\,\overrightarrow {{\tau_{h} }}$ is the joint moment acting at the hip joint.

Based on Eqs. (3) and (4), the relation between cable tension and joint moment for a cable can be written as:

5 $$\tau_{cable} = \left[ {\begin{array}{*{20}c} {\overrightarrow {{\tau_{h} }} } \\ {\overrightarrow {{\tau_{k} }} } \\ \end{array} } \right] = \left[ {\begin{array}{*{20}c} {J_{11} } \\ {J_{21} } \\ {J_{31} } \\ \end{array} } \right]F = J^{T} F$$

where $\tau_{cable}$ is the net joint moment induced due to applied cable tension *F* and is mapped by the Jacobian *J*.

For 5 cable configurations (Fig. 6a), each cable tension’s (*F*_*1*_,* F*_*2*_,* F*_*3*_,* F*_*4*_,* F*_*5*_) contribution to the joint moment is mapped using Jacobian vectors (*J*_*ij*_). The combination of all corresponding Jacobian results in a control performance matrix (*B*) that maps the cable tension to the net joint moment.

6 $$\begin{gathered} \tau_{cable} = B^{T} F,\,\,F = \left[ {\begin{array}{*{20}c} {F_{1} } & {F_{2} } & {F_{3} } & {F_{4} } & {F_{5} } \\ \end{array} } \right]^{T} \hfill \\ B = \left[ {\begin{array}{*{20}c} {J_{1} } & {J_{2} } & {J_{3} } & {J_{4} } & {J_{5} } \\ \end{array} } \right]^{T} = \left[ {\begin{array}{*{20}c} {J_{11} } & {J_{12} } & {J_{13} } & {J_{14} } & {J_{15} } \\ {J_{21} } & {J_{22} } & {J_{23} } & {J_{24} } & {J_{25} } \\ {J_{31} } & {J_{32} } & {J_{33} } & {J_{34} } & {J_{35} } \\ \end{array} } \right]^{T} \hfill \\ \,B \in {\mathbb{R}}^{5 \times 3} ,J \in {\mathbb{R}}^{3 \times 1} \hfill \\ \end{gathered}$$

Thus, the total joint moment in the Eq. (1) can be expressed as:

7 $$\tau = \tau_{cable} + \tau_{passive} = B^{T} F + \tau_{passive}$$

### Cable tension distribution and control

Maintaining cable tension is a crucial issue in cable-driven mechanisms. A minimum cable tension of 7N is set to ensure that the cable is always taut. At the same time, the maximum cable tension is limited to a level that keeps the maximum moment applied to the joint within a predefined range. Since C-LREX is designed as a human assistive device, the assistive moment it can provide is limited to moment equivalent to tension of 100N. The control performance matrix *B* may be square or rectangular depending on the number of cables used. Therefore, it may be challenging to estimate cable tension based on the required joint moment, and an optimization-based cable tension distribution approach is needed. Furthermore, depending on the number of cables employed, C-LREX could be an under-drive or over-drive system. In such cases, it’s difficult to guarantee a unique solution satisfying the system constraints. To guarantee a unique solution in all possible cases, a hybrid optimization problem (error minimization (primary) and control effort minimization (secondary)) was formulated and converted into QP (quadratic programming) to distribute the cable tensions to meet the joint moment requirements^8,45^.

The hybrid optimization problem is written as:

8 $$\left\{ \begin{gathered} \min \left( {\,\left\| {W_{v} \left( {B^{T} F - \tau } \right)} \right\| + \gamma \left\| {W_{F} F} \right\|} \right) \hfill \\ F_{\min } \le F \le F_{\max } \hfill \\ \end{gathered} \right.$$

In the above equation, *W*_*v*_ and *W*_*F*_ are weight factors assigned to primary and secondary optimization objectives respectively. *τ* represents the torque required in the model, and *B*^*T*^*F* represents the torque generated by the cables. These objectives are prioritized using weight $\gamma \,,\left( {0 < \gamma < 1} \right)$. The hybrid optimization problem is converted into a QP problem and is written as:

9 $$\left\{ \begin{gathered} \mathop {\min }\limits_{F} \left( {\frac{1}{2}F^{T} HF + f^{T} F} \right) \hfill \\ s.t.\,\left\{ {AF \le D} \right. \hfill \\ \end{gathered} \right.$$

where $$A = \left[ {\begin{array}{*{20}c} { - I} & I \\ \end{array} } \right]^{T} ,\,D = \left[ {\begin{array}{*{20}c} { - F_{\min } } & {F_{\max } } \\ \end{array} } \right]^{T}$$ *H* is the Hessian matrix and *f* is the gradient matrix calculated from the hybrid optimization function.

To control C-LREX, we implemented a three-layer control architecture based on impedance control, as previously described in reference^8^, to track healthy reference trajectory. Anthropometric data from Winter’s^46^, based on body weight and height, were adopted and the moments of inertia in the frontal and sagittal planes were kept the same. The reference trajectory used was based on Fukuchi et al. work^47^, which is the overground walking of a *3.48 s* gait cycle time.

### Cable routing constraints verification

When simulating an exoskeleton model, ensuring that the model’s cable routing meets certain requirements can be challenging. These requirements include:

1. The ability to generate both positive and negative joint moments at a given instant of motion,
2. A safe clearance distance between the cables and the user’s limb (especially at the hip and knee joints),
3. No intersection between cables in the routing space and,
4. Proper placement of the cuffs on the user’s limb segments to prevent interference between sets of cuffs.

Previous works^7^, ^19^, ^48^, ^32^ have imposed a wrench-closure workspace constraint to ensure a valid cuff location, but this is not sufficient since clearance and interference issues were not addressed. While some challenges can be manually tackled during simulation for specific configurations and routing, ensuring clearance and non-interference of cables during the entire gait cycle is difficult, especially for bi-planar models.

**Figure 7:**
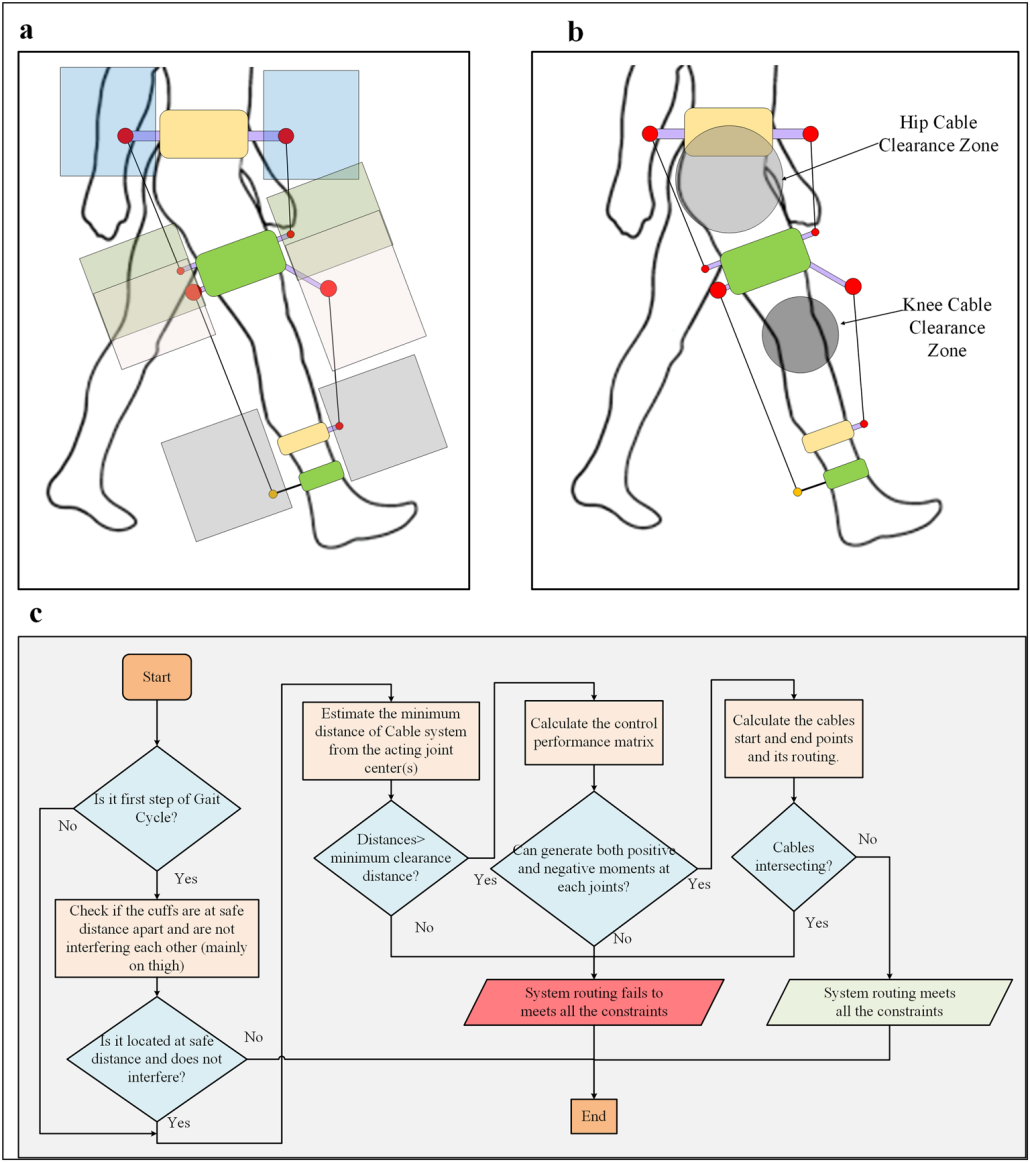
Cable routing constraint verification: (**a**) Typical zone for the cuff’s location during optimization (for 4 cables in the sagittal plane), (**b**) Clearance zone for cables for hip and knee joints. (**c**) Flowchart to estimate system capability to meet various routing constraints.

These challenges are more dominant when a single model uses optimal routing parameters for a given anthropometric data and reference trajectory as shown in Fig. 7a. Some cuffs can have overlapping zones, and, in such cases, it is important to ensure that the cuffs are located at a safe distance from each other and do not interfere with the cables.

To address these challenges, we introduced a framework to verify cable routing constraints. The routed cables must be placed at a safe distance from the user’s limb segment, with clearance zones defined as spheres in 3D space. This is to ensure that the cable will not interfere with the joints or lower limb segment. Furthermore, the framework is limited to estimating the interference between the cable and lower limb only. In our study conducted with stroke survivors using cable-driven robot C-ALEX^49^, the feasibility of walking without interference with the cables was demonstrated. While it is not advisable for participants to hold the handrails during walking as it interferes with the motor training experience, it may still be fine to occasionally do so on a treadmill^5,7^. However, the upper limb range of motion can be included in future in conjunction with upper limb rehabilitation device or incorporating upper limb trajectory information into the cable routing constraint verification module. Alternatively, the cuff zone for hip cuffs can be readjusted to avoid possibility of interference. The algorithm for verifying cable routing for C-LREX combines the above constraints and assumes that the limb’s physiological ROM (workspace) is achievable. If the cable routing fails to pass the verifications criteria at any instant of motion, the routing is either modified or ignored for further analysis, with modifications including changing the location of the bi-planar orientation of the cuffs. Figures 7a–c illustrate this process.

### Identification of ‘suitable’ configuration

**Figure 8:**
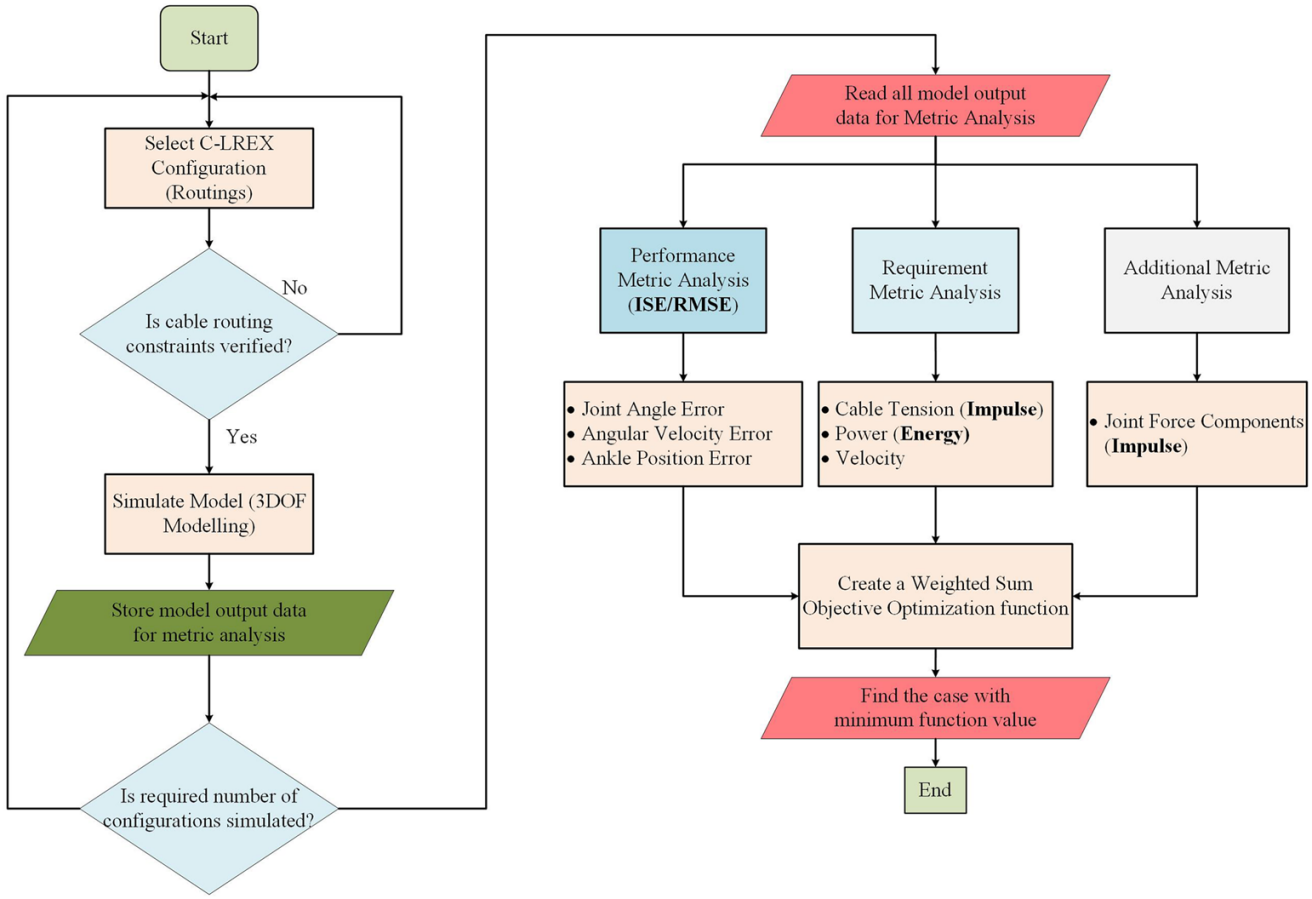
Flowchart depicting approach for Configurations simulation and Identification of optimal.

The various routing configurations were first tested via cable routing constraint verification. The successful configurations were then simulated using the 3DOF model mentioned earlier, and the outputs were stored for further comparison (as depicted in Fig. 8a). Various decision metrics^8^, including performance metrics such as tracking errors and requirement metrics such as cable tension, total power, etc. were analyzed from the model outputs. In our previous work^8^, the assignment of scores to each metric was carried out manually involving the division within a fraction of 100. This approach is preferrable when working with small sample sizes, as it allows for a comprehensive manual analysis of the results. When specific metrics need to be prioritized by varying their scores, it can lead to the discovery of novel optimal solutions. However, when dealing with larger samples, this manual process becomes tedious and time-consuming.

A weighted sum optimization function was then defined based on these decision metrics, where fixed weights were assigned to each metric. The objective was to identify the configuration with the minimum optimization function value (irrespective of the final function value) and select it as a “suitable” candidate (refer to the flowchart in Fig. 8b. However, changing the weight of the decision metric wouldn’t affect the optimal solution as similar weight will be applied to all conceptual models.

The weight sum optimization function, defined in Eq. (10), was formulated based on the metrics mentioned earlier. It is a combination of minimizing the error in trajectory tracking (via ISE—integral square error), minimizing the impulse required in C-LREX, minimizing the impulse exerted by C-LREX on lower limb joints, and minimizing the energy required in C-LREX to track the trajectory.

10 $$\begin{gathered} \min f \hfill \\ where,\,\,f = f_{1} + f_{2} + f_{3} + f_{4} \hfill \\ f_{1} = \lambda_{1} \left( {\int\limits_{0}^{\infty } {e^{2} \left( t \right)} \,\,dt} \right)\left( {\int\limits_{0}^{\infty } {e^{2} \left( t \right)} \,\,dt} \right)^{T} ,f_{2} = \lambda_{2} \left( {\int\limits_{0}^{\infty } {F\left( t \right)} \,\,dt} \right)\left( {\int\limits_{0}^{\infty } {F\left( t \right)} \,\,dt} \right)^{T} \hfill \\ f_{3} = \lambda_{3} \left( {\int\limits_{0}^{\infty } {F_{c} \left( t \right)} \,\,dt} \right)\left( {\int\limits_{0}^{\infty } {F_{c} \left( t \right)} \,\,dt} \right)^{T} ,f_{4} = \lambda_{4} \left( {\int\limits_{0}^{\infty } {P\left( t \right)} \,\,dt} \right)\left( {\int\limits_{0}^{\infty } {P\left( t \right)} \,\,dt} \right)^{T} \hfill \\ \end{gathered}$$

where *e* is the joint error, *F* is the cable tensions, *F*_*c*_ is joint component forces induced due to applied cable tension, *P* is the power demand in C-LREX configurations, and *λ*_*i*_ are weights assigned to each objective function. These weights can be altered based on preferences and constraints, and some objective functions can be excluded or included depending on specified conditions.

In Fig. 8, two key performance metrics are presented: ISE, which refers to Integral Square Error, and RMSE, representing Root Mean Square Error in trajectory tracking. The significance of these metrics lies in their ability to indicate the quality of trajectory tracking. Specifically, lower values of RMSE and ISE suggest a closer match of the desired trajectory, which higher values imply less accurate tracking. The impulse, which is calculated as the area under the force–time graph, serves the crucial purpose of optimizing cable tension and minimizing joint force components exerted over time. Cable tension, dependent on the cable’s routing, has a direct impact on the induced joint force components. Thus, minimizing cable tension is vital for the overall performance of the system. In the cable tension allocation algorithm Eq. (8), we have set a maximum limit on allowable cable tension within the system. However, the preference for optimizing impulse is geared towards reducing the allocation of higher cable tension over an extended period, rather than concentrating solely on peak cable tension. The deliberate choice to optimize for impulse, instead of peak cable tension, stems from our goal to minimize prolonged exposure to elevated force components on physiological joints throughout the gait cycle. By prioritizing impulse, a time-dependent measure, over mean cable tension, which remains time-invariant, our aim is to strike a balance in line with our objective of reducing the duration of higher joint force components. Furthermore, energy is assessed by computing the area under the power-time curve. This energy metric plays a key role in reducing the power requirement of the conceptual model, making it more efficient in tracking the healthy reference trajectory respectively.

### Supplementary Information

Supplementary Information.

## Data Availability

The datasets used and/or analyzed during the current study are available from the corresponding author on reasonable request.
