# Supplementary material for: Muscle-inspired bi-planar cable routing: a novel framework for designing cable driven lower limb rehabilitation exoskeletons (C-LREX)
Source: Sci Rep. 2024 Mar 2;14:5158. doi: 10.1038/s41598-024-55785-0 (PMC10908813; doi:10.1038/s41598-024-55785-0)
Supplement: Supplementary file 1 — Supplementary Information. [file 41598_2024_55785_MOESM1_ESM.docx]

**Supplementary Figures**

Table S- 1. Weights incorporated in weighted sum objective function.

| **Weights** | **Values** |
| --- | --- |
| λ_1_ | 10^5^ |
| λ_2_ | 10^-6^ |
| λ_3_ | 10^-6^ |
| λ_4_ | 10^-4^ |


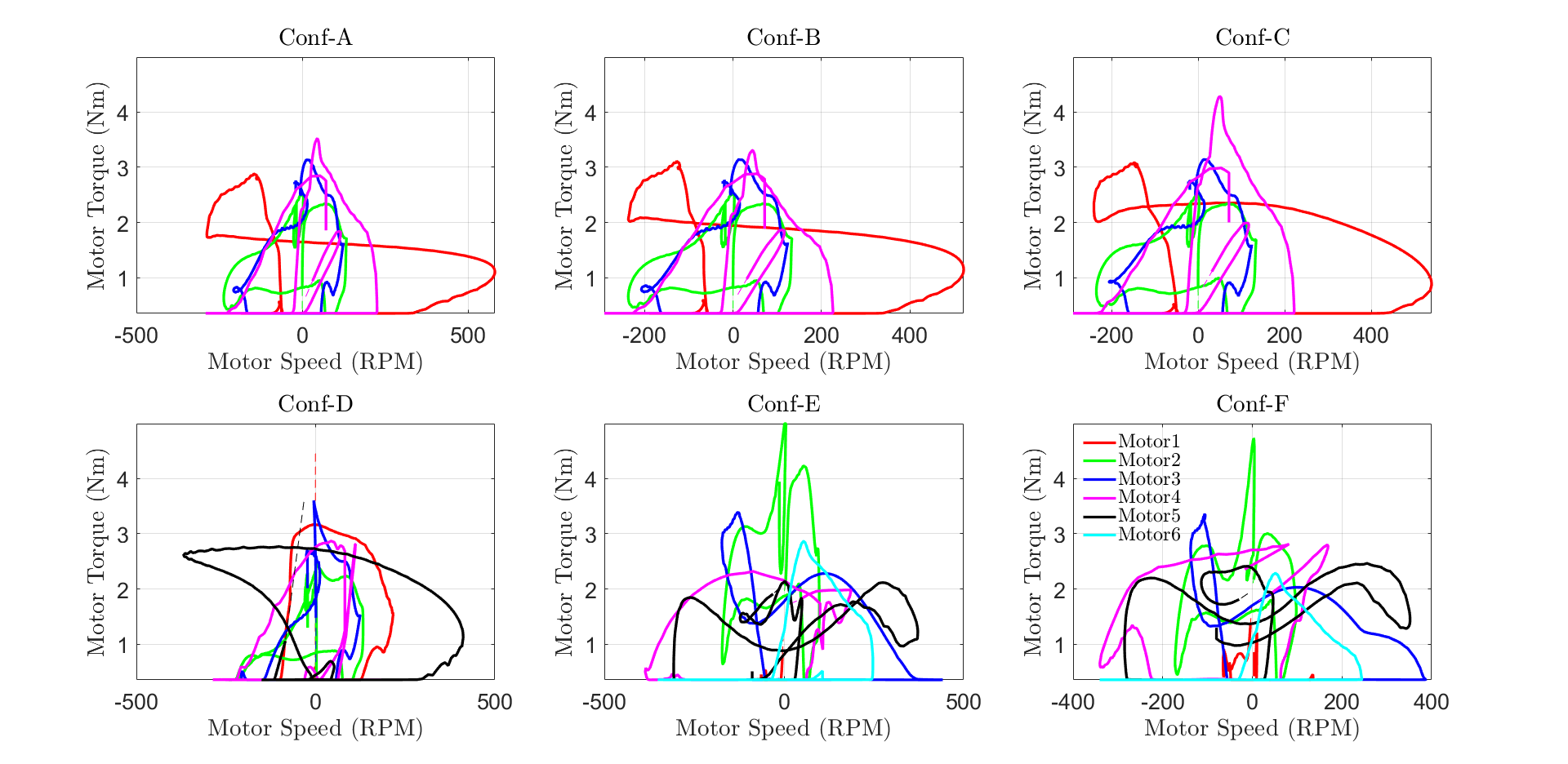


*Figure S- 1. Motor torque versus speed requirement in different configurations*


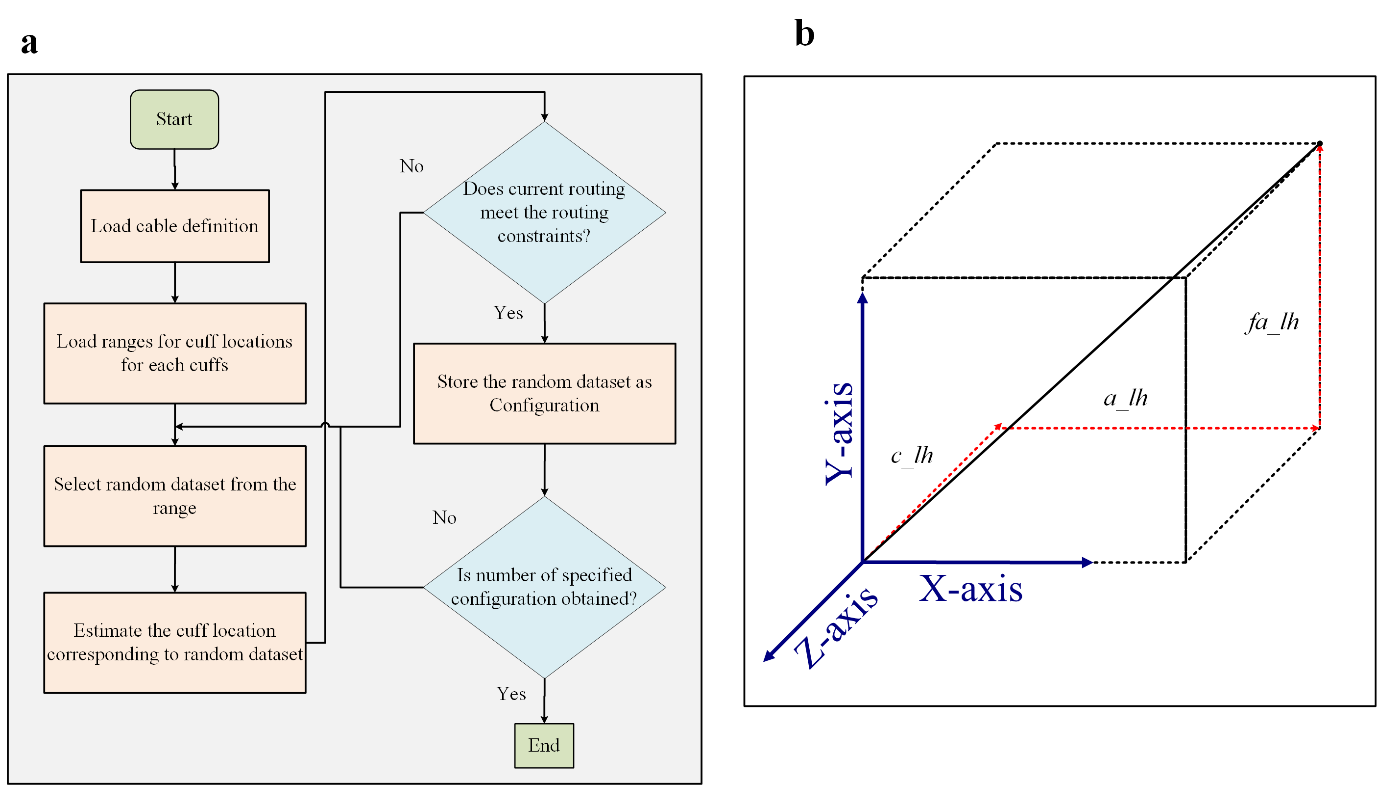


*Figure S- 2. Optimal routing parameter identification:* ***a.*** *Identification of feasible configurations from predefined cuff ranges,* ***b.*** *Cuff end location estimation along axes*

**Supplementary Data**

We have developed a MATLAB-based GUI application as supplementary material, aligned with the methodology described in the paper. This interactive tool is accessible to the research community and offers advanced functionalities for gait analysis and trajectory tracking with impaired gait. The GUI empowers designers by providing a user-friendly interface to customize various parameters, select tailored gait trajectory time, input user anthropometric information, and choose between sagittal plane-based or bi-planar-based lower limb modeling approaches.

The GUI application incorporates a wide range of features to support comprehensive gait analysis. Designers can input the hinge locations of each cuff and cable routings as inputs to the GUI, representing the conceptual model. Before proceeding to simulation, the application allows users to verify the configuration and ensures adherence to cable routing constraints, if selected to do so. Once simulated, the GUI provides diverse results, including trajectory tracking and cable tension requirements, which can be plotted for evaluation. Moreover, the GUI facilitates comparison of the results of different conceptual configurations, enabling quantitative analysis and generating comprehensive reports. Designers can benefit from dynamic tracking animations to visualize gait improvements and assess the effectiveness of tracking reference healthy trajectory with stroke gait.

The application also includes an optimal routing parameters identification section that utilizes Monte Carlo Simulation. By defining possible zones for cuff locations and specifying the number of samples to be analyzed, the GUI identifies the optimal hinge locations that optimize the performance while minimizing the requirements. The GUI application further supports the analysis of tracking reference healthy trajectory with stroke-impaired gait by incorporating subject-specific anthropometric information with the conceptual configuration. It allows designers to consider a wider range of impaired subjects and evaluate the feasibility of tracking reference healthy trajectory. With its intuitive interface and versatile functionalities, the GUI enhances the efficiency and accuracy of gait analysis and assistive analysis research.

We provide this MATLAB-based GUI application as supplementary material, aiming to facilitate reproducibility and promote further research in the field of gait analysis and trajectory tracking with impaired gait. The GUI empowers researchers to explore various configurations, analyze results quantitatively, and make informed decisions based on visual tracking animations. Its comprehensive features and user-friendly interface contribute to advancing personalized rehabilitation approaches for stroke-impaired gait.

The GUI and a demonstration to use it can be found at: <https://github.com/rajanprasad460/C-LREX-Tool>
